# MicroRNA expression profiling predicts clinical outcome of carboplatin/paclitaxel-based therapy in metastatic melanoma treated on the ECOG-ACRIN trial E2603

**DOI:** 10.1186/s13148-015-0092-2

**Published:** 2015-06-04

**Authors:** Liza C. Villaruz, Grace Huang, Marjorie Romkes, John M. Kirkwood, Shama C. Buch, Tomoko Nukui, Keith T. Flaherty, Sandra J. Lee, Melissa A. Wilson, Katherine L. Nathanson, Panayiotis V. Benos, Hussein A. Tawbi

**Affiliations:** University of Pittsburgh Cancer Institute, Pittsburgh, PA USA; University of Pittsburgh School of Medicine, Pittsburgh, PA USA; University of Pennsylvania, Philadelphia, PA USA; Massachusetts General Hospital, Boston, MA USA; Dana Farber Cancer Institute, Boston, MA USA; New York University, New York, NY USA

**Keywords:** Melanoma, MicroRNAs, Chemotherapy, Response, Biomarkers, Predictive

## Abstract

**Background:**

Carboplatin/paclitaxel (CP), with or without sorafenib, result in objective response rates of 18–20 % in unselected chemotherapy-naïve patients. Molecular predictors of survival and response to CP-based chemotherapy in metastatic melanoma (MM) are critical to improving the therapeutic index.

Intergroup trial E2603 randomized MM patients to CP with or without sorafenib. Expression data were collected from pre-treatment formalin-fixed paraffin-embedded (FFPE) tumor tissues from 115 of 823 patients enrolled on E2603. The selected patients were balanced across treatment arms, *BRAF* status, and clinical outcome. We generated data using Nanostring array (microRNA (miRNA) expression) and DNA-mediated annealing, selection, extension and ligation (DASL)/Illumina microarrays (HT12 v4) (mRNA expression) with protocols optimized for FFPE samples. Integrative computational analysis was performed using a novel Tree-guided Recursive Cluster Selection (T-ReCS) [1] algorithm to select the most informative features/genes, followed by TargetScan miRNA target prediction (Human v6.2) and mirConnX [2] for network inference.

**Results:**

T-ReCS identified *PLXNB1* as negatively associated with progression-free survival (PFS) and *miR-659-3p* as the primary miRNA associated positively with PFS. *miR*-*659*-*3p* was differentially expressed based on PFS but not based on treatment arm, *BRAF* or *NRAS* status. Dichotomized by median PFS (less vs greater than 4 months), *miR-659-3p* expression was significantly different. High *miR-659-3p* expression distinguished patients with responsive disease (complete or partial response) from patients with stable disease. *miR-659-3p* predicted gene targets include *NFIX*, which is a transcription factor known to interact with *c-Jun* and *AP-1* in the context of developmental processes and disease.

**Conclusions:**

This novel integrative analysis implicates *miR-659-3p* as a candidate predictive biomarker for MM patients treated with platinum-based chemotherapy and may serve to improve patient selection.

## Background

The incidence of melanoma has increased progressively over the last 30 years with an estimated 76,100 new cases in 2014 and a projected 9700 deaths (http://seer.cancer.gov/statfacts/html/melan.html). The prognosis of metastatic disease is historically poor, with 5-year survival rates of less than 6 % and median survival of 6 to 9 months [3, 4]. Until 2010, alkylating agents were the mainstay of treatment but associated with response rates less than 10–15 % [5, 6]. Platinum-based chemotherapy with carboplatin and paclitaxel (CP) is associated with response rates of 18–20 % in chemotherapy-naïve patients and 11–12 % in the second-line setting [7, 8]. Significant therapeutic advances have been made in recent years with FDA approval of the monoclonal antibody to CTLA-4, ipilimumab, which results in durable responses and improved overall survival (OS) in patients with metastatic melanoma (MM) compared with peptide vaccine or dacarbazine [9]. Antibody therapies against PD-1 and PD-L1 have also been associated with durable responses in MM, and the first agent targeting this pathway was granted FDA approval in September 2014 [10–12]. Finally, the selective targeting of *BRAF* activating mutations*,* which occur in about half of melanomas, with vemurafenib, dabrafenib, and trametinib, is associated with marked clinical activity and improved OS compared with chemotherapy [13, 14].

Despite the recent successes of immunotherapy and targeted therapy, chemotherapy continues to be a significant modality utilized worldwide in MM, especially after progression on immunotherapy and/or antitumor targeted agents. Recalcitrance of melanoma to chemotherapy is a significant issue due to limited response rates and lack of predictive biomarkers of chemotherapy benefit. Identification of biomarkers of response and survival for patients treated with chemotherapy is critical to allow more refined application of this modality and improving treatment outcomes. MicroRNAs (miRNAs) are endogenous non-coding RNAs approximately 22 nucleotides in length that regulate gene expression at the post-transcriptional level and play an important role in the regulation of tumor suppressor genes, oncogenes, and genes involved in the epigenetic machinery and cancer cell metastasis [15]. *miR*-*15a*, *miR*-*16*, and the *let*-*7* family function predominately as tumor suppressors, while the *miR*-*17*-*92* cluster functions as oncogenes [16–20]. Differential expression of four miRNAs (*miR-205*, *miR-23b*, *miR-146a*, and *miR-155*) distinguishes melanoma cell lines from nevi and primary or metastatic melanoma [19]. Differential miRNA expression also distinguishes among common melanoma histologic subtypes (superficial spreading melanoma vs nodular melanoma) and benign nevi compared with either primary or metastatic melanoma [21–24]. Of greatest importance, miRNAs have been shown to have prognostic implications in melanoma. Downregulation of *miR-125b* in patients with stage T2 primary cutaneous melanoma is associated with the presence of micrometastatic disease to the sentinel lymph node [25]. Analysis of primary and metastatic melanoma specimens shows that overexpression of an 18-miRNA signature is significantly correlated with longer survival (defined as a post-recurrence survival of more than 18 months) [26], indicating the value of miRNAs as candidate prognostic biomarkers. To date, there have been no studies examining miRNAs as candidate predictive biomarkers of response to chemotherapy in metastatic melanoma.

The Eastern Cooperative Oncology Group (ECOG) Study, E2603, was a phase III randomized controlled clinical trial evaluating the addition of the unselective multi-tyrosine kinase inhibitor, sorafenib, to platinum-based chemotherapy in patients with previously untreated MM. This phase III trial was the first to examine targeted therapy in melanoma but failed to meet its primary endpoint of demonstrating an OS benefit in MM patients treated with CP alone vs CP with sorafenib. The response rates and progression-free survival (PFS) were similar in either treatment arm. In the present study, our primary objective was to identify molecular predictors of survival and response to CP-based chemotherapy in the MM patients treated on E2603. We applied a novel method for cluster selection [1] to miRNA and gene expression data in this well-defined patient population in an effort to identify miRNAs as candidate biomarkers of prognosis and response to platinum-based chemotherapy. We then performed novel integrative analysis to construct a proposed genome-wide regulatory network, which lends insight into the role of our candidate miRNA biomarkers in the landscape of MM signaling. To our knowledge, our study is the first to utilize miRNAs as predictive biomarkers for therapy response in melanoma.

## Results and discussion

High-quality miRNA and mRNA expression data were generated from legacy samples from 115 MM patients treated on E2603. The clinicopathologic features for the patients included in this analysis are summarized in Table 1. The median PFS for this cohort of patients was 4.4 months and the median OS was 9.8 months.

**Table 1:** Characteristics of patients treated on E2603 included in this analysis

	*n* = 115
Characteristic	*n* (%)
Male sex	77 (67.0)
Median age, years (range)	59 (23–82)
AJCC stage	
Unresectable stage III	16 (13.9)
M1a/M1b	41 (35.7)
M1c	58 (50.4)
Serum LDH at baseline	
Normal	65 (56.5)
Above normal	47 (40.9)
ECOG performance status	
0	67 (58.3)
1	48 (41.7)
Prior systemic therapy	
None	52 (45.2)
Interferon, IL-2, GM-CSF	59 (51.3)
Investigational therapy	4 (3.5)
Treatment arm	
Carboplatin/paclitaxel	58 (50.4)
Carboplatin/paclitaxel/sorafenib	57 (49.6)
*BRAF* status	
Mutant	48 (41.8)
V600E	34 (29.6)
V600K	8 (7.0)
V600R	3 (2.6)
V600D	1 (0.9)
K601E	2 (1.7)
Wild-type	52 (45.2)
Unknown	15 (13.0)
*NRAS* status	
Mutant	22 (19.1)
G13R	2 (1.7)
G13C	1 (0.9)
Q61R	9 (7.8)
Q61K	8 (7.0)
D61L	2 (1.7)
Wild-type	78 (67.8)
Unknown	15 (13.0)
Survival	
Median OS, months (range)	9.8 (0.5–56.9)
Median PFS, months (range)	4.4 (0.5–42.1)

### PLXNB1 and RAD23 are associated with shortened PFS in metastatic melanoma

**Fig. 1:**
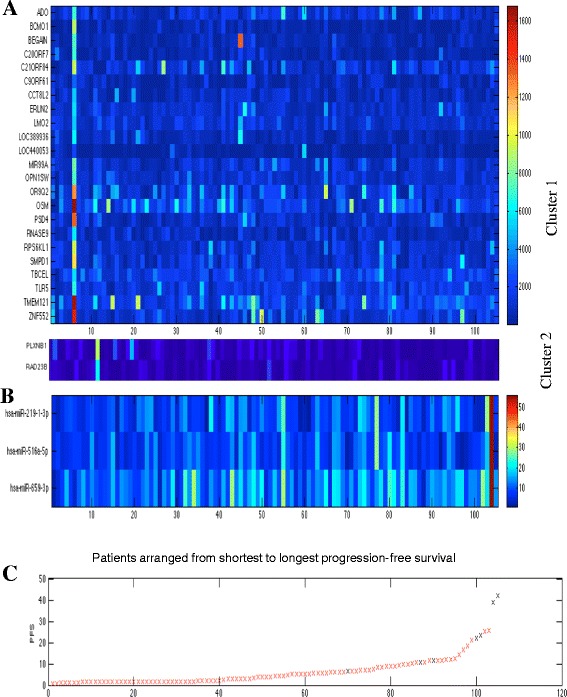
T-ReCS identified two major mRNA clusters (**a**) and one major cluster of three miRNAs (**b**) as predictive of progression-free survival (PFS), represented at heatmaps. Patients are ordered from shortest to longest progression-free survival (**c**)

Among the single genes selected with Tree-guided Recursive Cluster Selection (T-ReCS), *PLXNB1*, a known tumor suppressor of melanoma, had the most significant negative association with PFS (*p* = 0.0001; Fig. 1a) [27, 28]. T-ReCS found gene *RAD23* in the same group as *PLXNB1. RAD23*, a component of the nucleotide excision repair mechanism, was also negatively associated with PFS. In the other cluster, T-ReCS found *BCMO1*, which encodes a key enzyme in beta-carotene metabolism to vitamin A and is thus important for skin protection. *BCMO1* was part of a very significant group variable, which included *LOC389936*, a shRNA construct with no known association with melanoma.

### miR-659-3p is associated with improved PFS in metastatic melanoma

T-ReCS identified *miR-659-3p* as the primary miRNA associated positively with PFS (*p* = 0.008). The *miR-659-3p* cluster also included *miR-219-3p* and *miR-519-5p* (Fig. 1b), which had less significant or no association with PFS (*p* = 0.036 and *p* = 0.12, respectively). We examined the Cox regression coefficients of this selected cluster to interrogate our results. Indeed, all of these miRNAs were positively associated with PFS. *miR-659-3p* was not differentially expressed based on the treatment arm (CP with sorafenib vs CP without sorafenib; *p* = 0.6), *BRAF* mutation status (mutant vs wild-type; *p* = 0.34) or *NRAS* mutation status (mutant vs wild-type; *p* = 0.55). We dichotomized PFS less than 4 months vs greater than 4 months, which is the median PFS of patients treated with CP with or without sorafenib on E2603. Higher *miR-659-3p* expression remained prognostic of longer PFS (*p* = 0.03). Further partitioning of the dataset into terciles or quantiles did not yield any significant results, presumably due to sample size. We also noticed that higher *miR-659-3p* expression was generally observed in patients who achieved an objective response (complete or partial response) as opposed to patients with stable disease by Response Evaluation Criteria In Solid Tumors (RECIST) (*p* = 0.04.)

### miR-659-3p gene targets

**Fig. 2:**
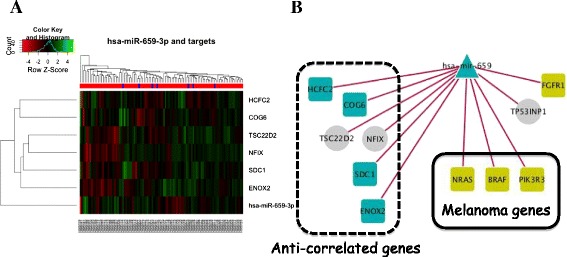
Clustergram of genes whose expression was inversely correlated with *miR-659-3p* expression in our dataset (**a**). Target predictions of *miR-659-3p* identified utilizing mirConnX software for network inference (**b**). Several oncogenes known to be involved in melanoma disease mechanisms are highlighted in *gold*. Genes are represented by *squares*, miRNAs by *triangles*, and transcription factors by *circles. Left panel*, anti-correlated genes; *center panel*, genes in the KEGG melanoma pathway; *top right corner*, other cancer-related genes

*miR-659-3p* gene targets were inferred from TargetScan Human v6.2 and from the mirConnX prior information file [29]. The list of predicted targets included genes relevant to the MAP kinase pathway (*BRAF* and *NRAS*), genes relevant to the PI3K/MTOR, FGFR, and RAC1 pathways, as well as genes involved in DNA repair (Table 2; Fig. 2b). However, the expression of most of those genes was not significantly anti-correlated with *miR-659-3p* expression. This might be explained by the fact that each individual miRNA target usually decreases the expression of the host gene by a small amount. However, we did find a significant anti-correlation (*p* < 0.01) between *miR-659-3p* and its predicted targets *COG6*, *ENOX2*, *HCFC2*, *NFIX*, *SDC1*, and *TSC22D2* (Fig. 2a). NFIX (nuclear factor 1 X-type), a member of the NF-1 family, is particularly important since it has been implicated in many developmental and biological processes and diseases, and the role of the NF-1 pathway is emerging as a potential therapeutic target [30–33]. Studies have also shown that it interacts with the oncogenes *c-Jun* and *AP-1* [34].

**Table 2:** Gene targets of interest of *miR-659-3p* identified using TargetScan Human V6.2

Relevant pathway	Gene target
MAPK	BRAF
	NRAS
	MAP7
	MAPK9
PI3K/MTOR	PIK3C2A
	FBXW2
	RICTOR
FGFR	FGF12
	FGF18
RAC1	RAC1
DNA repair	PARP16
	TP53INP1
	CDK19
	ABCC1

### miR-331-3p distinguishes OS in metastatic melanoma

Running T-ReCS with OS as the outcome variable, we found *miR-331-3p* as the primary miRNA positively associated with OS, while its cluster also included *miR-93-5p. miR-331-3p* was not differentially expressed based on the treatment arm (CP with sorafenib vs CP without sorafenib; *p* = 0.706), *BRAF* mutation status (mutant vs wild-type; *p* = 0.1909) or *NRAS* mutation status (mutant vs wild-type; *p* = 0.52).

## Conclusions

We have utilized a novel integrative analysis of miRNA and gene expression data to identify molecular biomarkers of PFS and response and found *miR-659-3p* expression to be positively associated with longer PFS, distinguishing patients with disease that is responsive to CP-based therapy. Characterization of the functional significance of *miR-659-3p* has been limited to the neuroscience literature, where it has been shown to bind a common genetic variant of and regulate the expression of the gene *progranulin*, which may significantly increase the risk for frontotemporal dementia [35]. The functional significance of *miR-659-3p* in cancer and melanoma is as of yet unknown. Of particular significance in our study is the identification of gene targets predicted for *miR-659-3p* and the construction of a proposed regulatory network which may lend insight into the mechanistic role of *miR-659-3p* in response to cytotoxic chemotherapy in melanoma.

Gene expression analysis showed *PLXNB1* to be associated with foreshortened PFS. *PLXNB1* has previously been shown to be lost in MM and in deeply invasive primary tumors and is strongly inhibited by MAP kinase signaling in melanoma cells and melanocytes [27, 28]. In preclinical models, plexin B1 suppressed proliferation, enhanced migration, stimulated Akt activation and, of particular interest, has been reported to render melanoma cell lines resistant to cisplatin-induced apoptosis [28]. Plexin B1 has been shown to inhibit c-Met in response to its ligand hepatocyte growth factor (HGF) and is predicted to be a classic tumor suppressor protein in melanomas in which progression is c-Met dependent [28]. *RAD23* was also identified in our model as associated with shorter PFS and is a component of the protein complex that specifically complements the nucleotide excision repair defect of xeroderma pigmentosum group C (XP-c) cell extracts in vitro [36]. Furthermore, *RAD23* has been shown to be downregulated in glutathione *S*-transferase M1 (GSTM1)-null melanomas in patients reporting histories of sunburns [37]. RAD23 has been implicated as a biomarker of response to epigenetic therapy, in particular HDAC inhibition, through a regulatory circuit that involves RAD23B and HDAC6 [38, 39].

Predicted gene targets of *miR-659-3p* selected in our analysis are genes important to the MAP kinase signaling pathway, in particular *BRAF* and *NRAS*, and genes relevant to the PI3K/MTOR, FGFR, and RAC1 pathways, as well as genes involved in DNA repair. BRAF, a member of the RAF kinase family of serine/threonine protein kinases, is mutated in 40–50 % of melanomas and results most frequently from a valine (V) to glutamic acid (E) substitution at residue 600 (*BRAF*^*V600E*^), rendering mutant BRAF protein which no longer requires dimerization for its activity, and is highly predictive of therapeutic response to the specific BRAF inhibitors, vemurafenib and dabrafenib. Mutations in *NRAS* occur in 15–20 % of melanomas and result in constitutive activation of the NRAS protein and enhanced MAP kinase signaling [40–42]. FGFR1 signaling has been implicated in melanoma progression [43]; introduction of antisense oligonucleotides targeted toward *FGFR1* into metastatic cell lines results in decreased proliferation and signs of differentiation [44, 45]; and injection of an antisense *FGFR1* construct into primary and metastatic melanomas grown in nude mice results in inhibition of tumor growth and induction of apoptosis [46, 47]. RAC1, a member of the Rho family of GTPases, is involved in cellular proliferation, survival, motility, and invasiveness [48]. The *RAC1* mutation resulting in the substitution of proline for serine at residue 29 is the most frequently mutated amino acid in MM, after *V600* mutations in *BRAF* and the glutamine (Q) substitution at position 61 in *NRAS. RAC1* mutations are observed in 4 % of all melanomas and 9 % of sun-exposed melanomas and may occur either independently or in conjunction with *BRAF* and *NRAS* mutations, suggesting growth advantages distinct from activation of the MAP kinase signaling pathway [49, 50]. RAC1 and other members of the Rho family of GTPases have been implicated as key modulators of sensitivity to DNA-damaging agents; in particular, chemical inhibition of RAC1 has been shown to sensitize drug-resistant ovarian cancer cell lines to cisplatin [51–53].

While the association between *miR-659-3p* and these predicted genes targets is intriguing, it is important to note that our gene expression data do not show strong correlations with *miR-659-3p* and at this point, these findings are not confirmed. These findings do, however, suggest biological relevance of *miR-659-3p* in the context of known oncogenic drivers in melanoma, an association which requires further investigation in future studies. We did find a significant anti-correlation between *miR-659-3p* and a small set of genes. *NFIX*, which has been implicated in many developmental and biological processes and diseases, is one of them and has been shown to interact with known oncogenes. This interaction may represent a clue as to functional relevance of *miR-659-3p* and requires further validation.

Our study identified an association between *miR-659-3p* and the clinical activity of CP-based therapy implicating *miR-659-3p* as a candidate predictive biomarker in patients with metastatic melanoma. This analysis did not include untreated MM patients, and therefore, we are unable to evaluate the prognostic value of *miR-659-3p*, i.e., we are unable to assess if the association between *miR-659-3p* and PFS persists in the absence of therapy. These data were generated from formalin-fixed paraffin-embedded (FFPE) legacy samples from a phase III multicenter clinical trial that represents a limitation of this study. Recovery of RNA from archival FFPE tissues can be challenging due to strand breakage and cross-linking by formalin fixation, which may inhibit polymerase in PCR-based molecular assays. We overcame this challenge by utilizing technology optimized for FFPE samples. Specifically, the miRNA expression assay used in this study allows for direct digital detection and counting of miRNAs in a single reaction without the need for an amplification step. Another limitation is that this bio-analysis represents a subset of the 823 patients enrolled on E2603. Archival tissue was not compulsory in E2603, and therefore, genotyping information is available for only 179 patients treated on this trial. It is notable that the patients included in this analysis were similar to the overall E2603 population and the population of patients for whom genotyping information is available [7, 54].

This novel integrative analysis suggests *miR-659-3p* as a candidate biomarker of response in MM patients treated with platinum-based chemotherapy and may serve to improve patient selection. To our knowledge, our study is the first to identify a miRNA species as a predictor of therapy outcome in metastatic melanoma. While our proposed regulatory network embeds *miR-659-3p* in the landscape of melanoma signaling, functional studies are paramount to the validation of this miRNA as a mediator of melanoma signaling. The value of the proposed regulatory network is to inform upon pathways which should be interrogated as we continue to elucidate the role of *miR-659-3p* as a predictive biomarker. Independent validation of the predictive value of *miR-659-3p* is currently ongoing in an independent cohort of MM patients treated with carboplatin and paclitaxel.

## Methods

### Patients and study design

E2603 enrolled patients with pathologically confirmed advanced unresectable or metastatic melanoma without brain involvement, who had no previous treatment with chemotherapeutic or MAPK pathway targeting agents. The study design has been previously described [7]. Key inclusion criteria were the presence of measurable disease by RECIST version 1.0, at least 18 years of age, ECOG performance status of 0 or 1, and satisfactory baseline organ function. Pre-treatment specimen collection was conducted as part of the clinical trial and analyzed with institutional review board approval. From the 823 patients enrolled on the E2603 clinical trial, 620 archival pre-treatment samples were available for analysis. One hundred and twenty pre-treatment formalin fixed paraffin-embedded (FFPE) tumor tissues from 115 patients were selected to ensure a balanced representation of patients based on treatment arm, *BRAF* status, and clinical outcome (PFS less than 4 months vs greater than 4 months) were included in this analysis.

### Treatment and assessment of response

The patients were randomized in a double-blind fashion to receive CP, either with or without sorafenib. Paclitaxel was administered at 225 mg/m^2^ over 3 h followed by carboplatin AUC 6 IV over 30 min on day 1 of each 21-day cycle during cycles 1–4. During cycles 5 through 10, paclitaxel was administered at 175 mg/m^2^ IV over 3 h followed by carboplatin AUC 5 IV over 30 min. Sorafenib 400 mg or placebo was administered twice daily by mouth on days 2 through 19 of each 21-day cycle. Upon completion of 10 cycles of chemotherapy, sorafenib 400 mg twice daily by mouth or placebo was administered daily until disease progression or intolerable toxicity. Tumor response was assessed every 2 cycles during cycles 1 through 10 and then every 3 cycles. Response was defined by RECIST version 1.0.

### Mutation profiling

Genotyping was performed using a custom iPlex Sequenom panel interrogating 74 mutations in 13 genes in the Perelman School of Medicine Molecular Profiling Facility, as previously described [54].

### RNA extraction

Total RNA was isolated from melanoma tissues using the PerfectPure RNA Tissue kit (5Prime Inc., MD, USA) at the University of Pittsburgh Cancer Institute (UPCI). RNA was quantified using Ribogreen RNA quantitation Kit (Molecular Probes, Eugene OR). RNA quality was also evaluated by RNA integrity number using the Agilent Bioanalyzer.

### miRNA microarray expression profiling and gene expression analysis

Profiling of miRNA levels was performed at the UPCI utilizing technology optimized for FFPE samples: Nanostring technology (Ncounter Human v2 miRNA Expression Assay) for miRNA analysis of 800 human miRNAs was derived from miRBase v.18. Total RNA was used as input for nCounter miRNA sample preparation reactions. All sample preparation and processing were performed according to the manufacturer’s protocol. Following ligation, sample preparation reactions were purified and diluted, and hybridization reactions were performed by incubation at 65 °C for a minimum of 18 h. Hybridized probes were purified and counted on the nCounter Prep Station and Digital Analyzer (NanoString) following the manufacturer’s instructions. For each assay, a high-density scan (600 fields of view) was performed. Whole-genome gene expression analysis was also carried out using DNA-mediated annealing, selection, extension, and ligation (DASL)/Illumina HT-12 v4 Expression BeadChip, a technology optimized for FFPE samples, at the UPCI according to the manufacturer’s protocol.

### Statistical analysis for mRNA and miRNA biomarkers predictive of survival

We performed feature selection at the cluster level utilizing a novel algorithm (T-ReCS) [1]*.* Briefly, starting from a standard feature selection method against a target variable (in our case, PFS or OS), T-ReCS expands the single features into clusters of features according to two statistical criteria that maintain the prediction accuracy. The clusters are defined dynamically as groups of similarly expressed genes. T-ReCS works in two steps. First, it partitions the feature space into a tree structure with the root being the set of all variables (mRNA or miRNA genes, respectively), while each leaf is a single variable. Each internal node in the tree represents a cluster of variables, its descendants, whose values (expression) are similar. The lower in the tree a node is, the more similar the patterns of its members are. For our implementation, we use the tree produced by our Recursive K-means Spectral Clustering (ReKS), which was previously developed for heterogeneous clinical data [29]. The second step of T-ReCS is feature selection operating on the tree structure iteratively. Starting with a set of variables (tree leafs) selected with any feature selection method, it ascends the tree one level at a time and statistically assesses whether the group variables (internal nodes) retain the same predictive properties. For statistical assessment, T-ReCS uses two conditional independence tests with *p* value thresholds determined by cross-validation. For survival target variables, T-ReCS uses survival MMPC (sMMPC) [55] for single-variable selection. The association between selected genes and survival was tested using Cox regression (Cox proportional hazards model) [56].

### Regulatory associations between predictive mRNA and miRNA features

A regulatory network was constructed from the mRNA and miRNA genes and their clusters that were selected as characteristic of PFS. The mRNA targets of miRNAs were predicted using TargetScan Human v6.2 [57], while transcription factor (TF)→gene and TF→miRNA regulatory interactions were obtained from the prior (static) network of mirConnX [2]. mirConnX is a user-friendly web interface for inferring, displaying, and parsing mRNA and miRNA gene regulatory networks. mirConnX infers regulatory networks by combining expression correlation values (dynamic network) with regulatory interactions obtained from the literature and from computational predictions (static network).

## Abbreviations

CP: carboplatin/Paclitaxel
FFPE: formalin-fixed paraffin-embedded
MM: metastatic melanoma
OS: overall survival
PFS: progression-free survival
